# Progress Toward Eliminating Mother to Child Transmission of HIV in Kenya: Review of Treatment Guideline Uptake and Pediatric Transmission at Four Government Hospitals Between 2010 and 2012

**DOI:** 10.1007/s10461-015-1071-5

**Published:** 2015-04-23

**Authors:** Sarah Finocchario-Kessler, Kristine F. Clark, Samoel Khamadi, Brad J. Gautney, Vincent Okoth, Kathy Goggin, HITSystem Study Team

**Affiliations:** 1 Department of Family Medicine, University of Kansas Medical Center, Kansas City, KS USA; 2 Department of Global Studies, University of Kansas, Lawrence, KS USA; 3 Kenya Medical Research Institute, Centre for Virus Research, Nairobi, Kenya; 4 Global Health Innovations, Kansas City, USA; 5 Health Services and Outcomes Research, Children’s Mercy Hospital, Kansas City, USA

**Keywords:** Preventing mother to child transmission (PMTCT), Eliminating pediatric infection, HIV, WHO guidelines, Option B+, Gestational week of initiation, Adherence, Retention in care

## Abstract

We analyzed prevention of mother-to-child transmission (PMTCT) data from a retrospective cohort of n = 1365 HIV+ mothers who enrolled their HIV-exposed infants in early infant diagnosis services in four Kenyan government hospitals from 2010 to 2012. Less than 15 and 20 % of mother-infant pairs were provided with regimens that met WHO Option A and B/B+ guidelines, respectively. Annually, the gestational age at treatment initiation decreased, while uptake of Option B/B+ increased (all p’s < 0.001). Pediatric HIV infection was halved (8.6–4.3 %), yet varied significantly by hospital. In multivariable analyses, HIV-exposed infants who received no PMTCT (AOR 4.6 [2.49, 8.62], p < 0.001), mixed foods (AOR 5.0 [2.77, 9.02], p < 0.001), and care at one of the four hospitals (AOR 3.0 [1.51, 5.92], p = 0.002) were more likely to be HIV-infected. While the administration and uptake of WHO PMTCT guidelines is improving, an expanded focus on retention and medication adherence will further reduce pediatric HIV transmission.

## Resumen

Se analizaron los datos de prevención de la transmisión materno-infantil (PTMI) de un cohorte retrospectivo de n = 1.365 VIH+ madres que inscribieron a sus bebés expuestos al VIH en los servicios de Diagnóstico Temprano Infantil (Early Infant Diagnosis EID) en cuatro hospitales del gobierno de Kenia del 2010-2012. Menos del 15% y el 20% de parejas madre-hijo fueron provistos de regímenes que cumplieron con las directrices de la OMS (Organización Mundial de la Salud) opción A y B/B +, respectivamente. Anualmente, la edad gestacional al momento del inicio del tratamiento disminuyó, mientras que la absorción de la Opción B/B + aumentó (todo de p <0,001). La infección pediátrica del VIH se redujo a la mitad (8,6% a 4,3%), pero ha variado significativamente por hospital. En los análisis multivariable, los bebés expuestos al VIH que no recibieron la PTMI (AOR 4,6 [2,49, 8,62], p <0,001), alimentos mixtos (AOR 5,0 [2,77, 9,02], p <0,001), y la atención en uno de los cuatro hospitales (AOR 3,0 [1,51, 5,92], p = 0,002) tenían más probabilidades de estar infectados por el VIH. Mientras que la administración y el seguimiento de las normas de PTMI de la OMS están mejorando, un enfoque ampliado sobre la retención y adherencia a la medicación reducirá aún más la transmisión del VIH pediátrico.

## Introduction

Despite a consorted effort to expand access to HIV testing in antenatal care (ANC) and antiretroviral (ARV) prophylaxis for HIV+ pregnant women [1, 2], Kenya continues to struggle to provide comprehensive HIV prevention and treatment services for women and infants. In 2012, an estimated 86,000 (76,000–97,000) pregnant women in Kenya were living with HIV [3]. Among HIV-positive mothers, 53 % [47–60 %] received ARV prophylaxis during pregnancy, however; an estimated one-third received an inadequate or inferior ARV regimen [1–3]. Roughly 13,000 (10,000–17,000) [3] children ages 0–14 were newly infected in 2012. Furthermore, only 39 % [34–44 %] [4] of HIV-exposed infants receive timely virologic testing by 6 weeks of age in accordance with World Health Organization (WHO) guidelines.

In 2010, the WHO revised global guidelines for the prevention of mother-to-child transmission of HIV (PMTCT) outlining two options: A and B [5]. Option A was a short-term prophylaxis of zidovudine during pregnancy and single dose of nevirapine and zidovudine + lamivudine at delivery plus six additional daily doses of zidovudine + lamivudine for the mother and daily nevirapine syrup for infants throughout breastfeeding. Option B was maternal triple-drug ARV regimens throughout pregnancy and breastfeeding and daily infant nevirapine or zidovudine until 4–6 weeks postpartum. Both options were recommended for HIV+ pregnant women whose CD4 count or clinical staging did not otherwise make them eligible for treatment (<350 cells/mm^3^ or clinical stage 3 or 4). The target for antenatal ARV initiation was also shifted from 28 to 14 weeks gestation [5]. In January 2010 the Kenya National AIDS & STI Control Programme (NASCOP) scaled up their PMTCT services to support WHO Option A [6].

**Fig. 1:**
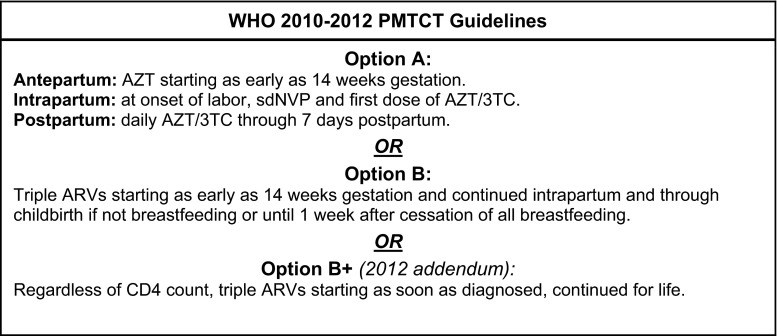
Explanation of WHO PMTCT Guidelines. *Note* Adapted from [10, p. 2]

In response to the overwhelming evidence of the benefits of early treatment initiation [7, 8], negative impact on mothers’ health of stopping ARVs postpartum, and global goals to eliminate new infections among children [9], new updated PMTCT guidelines were introduced in 2012. These updated guidelines introduced Option B+ which calls for ART initiation for all pregnant women upon HIV+ diagnosis, regardless of CD4 count, with sustained lifelong treatment [10]. These guidelines also strongly promoted immediate initiation of Option B/B+ and clarified their superiority over Option A. In response, in 2012, Kenya called for a transition to WHO Option B+ with the goal of national implementation by 2014 [4, 11]. See Fig. 1 for regimen and initiation period for each guideline.

Despite concerted efforts to provide treatment that is consistent with guidelines, clinical and patient-related barriers continue to negatively impact implementation. Clinical and system level barriers include inadequate and inconsistent training of clinical staff that results in delayed implementation of new guidelines [12–14], provision of less efficacious ARV regimens [12], and weak systems to support prospective patient follow up and retention [12, 14–16]. Many patients initiate treatment late and fail to maximize ANC (only 50 % of Kenyan women complete the four or more recommended antenatal appointments) [17]. Furthermore, poor medication adherence during pregnancy compromises the efficacy of medications [18, 19], and a multitude of factors (e.g., limited male partner support, resource constraints, and HIV-stigma) all contribute to incomplete retention in PMTCT and EID services [12, 14–16, 20]. Maintaining maternal support for treatment adherence is critical throughout the postpartum and breastfeeding periods, as only half of HIV+ mothers referred for ART after delivering their child actually follow-up on their own care [21].

To better understand recent trends and target future efforts in Kenya, this paper examined prescribing adherence to the 2010 WHO PMTCT guidelines and progress made toward 2012 Option B/B+ recommendations at four government hospitals. We assessed the proportion of mothers who received PMTCT, whether or not their treatment met WHO guidelines in regard to gestational week of treatment initiation and treatment regimen, and subsequent pediatric HIV infection. Given Kenya’s goal to achieve consistent nationwide WHO Option B+ implementation by 2014 and reduce perinatal transmission to <5 % by 2015, these data provide insight into current implementation practices and areas to target for improvement.

## Methods

We conducted a retrospective cohort analysis among 1365 HIV+ mothers who enrolled their HIV-exposed infants in early infant diagnosis (EID) services at four government hospitals that were piloting the HIV infant tracking system (HITSystem) intervention between April 2011 and December 2012. The HITSystem is an online intervention implemented at health facilities and central laboratories to maximize efficiency of HIV DNA PCR testing, and improve communication of results to mother/guardians, linkage to HIV treatment, and retention in EID. A detailed description of the HITSystem intervention and its impact have been previously published [22]. De-identified data regarding mothers’ antenatal PMTCT history, infant feeding method and postnatal ARV prophylaxis, the timing of infant testing, and infant HIV status were collected through the HITSystem. Only hospitals implementing the HITSystem during the specified review period were included. Mother-infant pairs were located in both peri-urban hospitals in Western Kenya (Hospitals A and B, both district level hospitals) and urban Nairobi health facilities [Hospitals C (high volume facility in a densely populated and underserved area) and D (referral maternity hospital)] with EID volumes ranging from 8.5 to 21.2 HIV-exposed infants per month.

## Procedures

As per the existing protocol at each of the hospitals, data regarding mothers’ PMTCT history were collected from mother’s medical file at the time of enrolling their infant in EID care. Maternal and infant data were collected through the HITSystem electronic database for the duration of the infant’s enrollment in EID care. Mothers’ were informed about the HITSystem and provided oral informed consent or declined participation (<1 %, recorded in enrollment notebook), in which case their information was collected in the EID Registry (standard of care). Participation involved minimal risk, and a signed consent form would have been the only document outside of protected medical records that would have linked the participant to their HIV+ serostatus. All patient records/information was anonymized and de-identified prior to analysis. Detailed methods regarding the implementation and data collection strategy for the HITSystem have been previously published [22]. The study protocol was approved by the Institutional Review Board at the Kenya Medical Research Institute (KEMRI) and the University of Kansas Medical Center.

## Measures

### PMTCT Treatment Guidelines

To examine provider adherence to WHO guidelines, we modified an established method for coding PMTCT guideline adherence described by Azcoaga-Lorenzo and colleagues [14]. Specifically, data for each mother-infant pair was coded as: (1) no intervention (no ARV prescribed), (2) late treatment initiation (correct ARV regimen combination prescribed later than WHO guidelines recommend), (3) initiation of WHO Option A, or (4) initiation of WHO Option B/B+. All recorded ARV regimens met the treatment criteria for either WHO Option A or WHO Option B/B+. Reported rates of adherence to guidelines reflect the proportion of providers that prescribed treatment regimens in perfect agreement with WHO PMTCT guidelines. For this study, we have opted to use the ‘Option B/B+’ categorization given the fact that WHO 2012 guidelines and the Kenya government endorse Option B+. However, the examination of life-long provision of Option B+ was not possible as long-term maternal follow-up was beyond the scope of the study.

### Trends in PMTCT

In order to determine year that PMTCT was accessed, mother-infant pairs were assigned to a calendar year of treatment based on their infant’s date of birth and an assumed 40 week gestation period. Specifically, pairs were assigned to the year in which the majority of the final 30 weeks of gestation occurred (i.e., 1 = 2010, 2 = 2011, 3 = 2012).

To examine trends in PMTCT regimen administration, we calculated the proportion receiving each regimen type (i.e., late, WHO A or WHO B/B+) over the total number of pairs who were prescribed any PMTCT. To examine trends in the timing of treatment initiation, we determined the gestational week when PMTCT was initiated for all pairs that received any PMTCT. We then compared the average gestational week of initiation across year of enrollment.

We calculated the proportion of infants who received postnatal prophylaxis: (1) NVP for 6 weeks, (2) NVP for 6 months, or (3) other treatment, over the total number of infants whose mother’s were prescribed PMTCT. Finally we calculated the proportion of all HIV-exposed infants who received cotrimoxazole in the context of EID services.

### Pediatric HIV Transmission and Associated Variables

Infant test results were coded as ‘0’ if HIV-negative, ‘1’ if HIV-positive, and ‘2’ if the test result was indeterminate. Positive HIV PCR results were calculated among all infants with a PCR test result. We compared the proportion of HIV-positive results for each year of enrollment to examine trends in infant HIV transmission. Infant age at HIV DNA PCR testing was calculated by comparing infant date of birth to date of dried blood spot collection and analyzed to report median age (in weeks). Other clinically relevant variables that could affect transmission e.g., infant feeding method at the time of testing (exclusive breast feeding, replacement feeding, and mixed feeding), and postnatal prophylaxis [ART (NVP for 6 weeks or 6 months) and cotrimoxazole to prevent pneumocystis carinii pneumonia] were collected from the HITSystem.

Hospital was included as a covariate to assess the impact of the health facility where mother-infant pairs received care. We assigned arbitrary titles (e.g. Hospital A, B etc.) to each hospital rather than reporting them by name.

## Analyses

Data from each hospital were cleaned and coded in separate excel files generated by the HITSystem before being merged into SPSS© version 20 for analysis. Frequencies and percentages were calculated for categorical variables such as PMTCT treatment regimen and infant HIV infection, and measures of central tendency and variability (mean, median, range, and standard deviation) were examined for continuous variables such as week of PMTCT treatment initiation and infant age at first PCR test. An ANOVA was used to determine if the week that mothers initiated PMTCT treatment varied across the years examined. Significant omnibus results were followed-up with Tukey HSD post hoc analyses to determine which years were significantly different from others. We used χ^2^ statistics to explore trends in the proportions of pairs receiving Option B/B+ and MTCT rates each year. Similarly, χ^2^ tests were used to determined significant differences in HIV transmission by type of PMTCT regimen. We conducted bivariate and multivariable logistic regression analyses to assess associations between independent variables (PMTCT regimen, hospital, infant feeding method, and postnatal prophylaxis) and pediatric HIV transmission.

## Results

PMTCT regimen was documented for 1134 (83.1 %) mothers enrolled in EID at the study sites. Rates of mothers who received some form of ARV therapy for PMTCT were consistently higher in the urban versus peri-urban sites. The majority of HIV+ pregnant women (66.3 %) were prescribed a PMTCT regimen late (≥14 gestational weeks). The most commonly prescribed regimens prior to 15 weeks was ART in accordance with WHO Option B/B+ guidelines (at Hospitals A, B, and D). AZT prescribed in accordance with WHO Option A was the most commonly prescribed regimen at Hospital C. Infants’ first HIV PCR test occurred at a median age of 6.8 (IQR 6.1) postnatal weeks when the majority (84.4 %) were exclusively breastfed. Administration of ART postnatal prophylaxis was high across sites (92.8 %), with over half (54.8 %) prescribed daily NVP for 6 months compared to the shorter duration of 6 weeks (42.1 %). Table 1 displays a breakdown of the prescribed PMTCT regimen, postnatal prophylaxis, HIV testing, and feeding method by hospital.

**Table 1:** Description of sample by hospital

Hospital	A	B	C	D	Total
Mother-infant pairs	203 (14.9 %)	324 (23.7 %)	287 (21.0 %)	551 (40.4 %)	1365 (100 %)
PMTCT regimen					
Total of mothers on any PMTCT	128/203 (63.1 %)	214/324 (66.0 %)	263/287 (91.6 %)	529/551 (96.0 %)	1134/1365 (83.1 %)
Late PMTCT Initiation	69/128 (53.9 %)	78/214 (36.4 %)	144/263 (54.8 %)	461/529 (87.1 %)	752/1134 (66.3 %)
WHO Option A	27/128 (21.1 %)	46/214 (21.5 %)	73/263 (27.8 %)	14/529 (2.6 %)	160/1134 (14.1 %)
WHO Option B/B+	32/128 (25.0 %)	90/214 (42.1 %)	46/263 (17.5 %)	54/529 (10.2 %)	222/1134 (19.6 %)
Infant postpartum prophylaxis					
Received ART prophylaxis	176/203 (86.7 %)	275/324 (84.9 %)	271/287 (94.4 %)	545/551 (98.9 %)	1267/1365 (92.8 %)
NVP for 6 weeks (only)	77/176 (43.8 %)	43/275 (15.6 %)	176/271 (64.9 %)	237/545 (43.5 %)	533/1267 (42.1 %)
NVP for 6 months	98/176 (55.7 %)	230/275 (83.6 %)	92/271 (33.9 %)	274/545 (50.3 %)	694/1267 (54.8 %)
Other regimen^a^	1/176 (0.6 %)	2/275 (0.7 %)	3/271 (1.1 %)	34/545 (6.2 %)	40/1267 (3.2 %)
Received cotrimoxazole	203/203 (100.0 %)	319/324 (98.5 %)	286/287 (99.7 %)	551/551 (100.0 %)	1359/1365 (99.6 %)
Infant HIV testing					
Infants tested for HIV	203/203 (100.0 %)	313/324 (96.6 %)	283/287 (98.6 %)	548/551 (99.5 %)	1347/1365 (98.7 %)
Median infant age (in weeks) at first PCR	6.64 SD 17.71	9.00 SD 15.74	6.57 SD 12.57	6.57 SD 13.04	6.86 SD 14.41
Infants testing HIV+	19/203 (9.3 %)	18/313 (5.7 %)	28/283 (9.9 %)	14/548 (2.5 %)	79/1347 (5.9 %)
Infants with an indeterminate HIV test result	1/203 (0.5 %)	4/313 (1.3 %)	1/283 (0.3 %)	0/548 (0 %)	6/1347 (0.4 %)
Infant feeding method					
Breastfeeding Only	168/195 (86.2 %)	238/299 (79.6 %)	252/285 (88.4 %)	464/551 (84.2 %)	1122/1330 (84.4 %)
Replacement feeding only	10/195 (5.1 %)	19/299 (6.4 %)	4/285 (1.4 %)	61/551 (11.1 %)	94/1330 (7.1 %)
Mixed feeding	17/195 (8.7 %)	42/299 (14.0 %)	29/285 (10.2 %)	26/551 (4.7 %)	114/1330 (8.6 %)

^a^Infant postpartum regimens were combined into the “other” category based on their small percentages. These regimens include “AZT for 6 weeks” N = 5; “single-dose nevirapine” N = 3, and “AZT + 3TC QID × 1 week” N = 32. Infant feeding method was not available for all pairs

### Provider Adherence to PMTCT Guidelines

Overall, less than 20 % of mother-infant pairs were provided with services that met WHO 2010 Option B/B+ guidelines (i.e., ART commencing at 14 weeks gestation). Hospital B experienced the highest rate of WHO 2010 Option B/B+ guideline-consistent services, range 10 % (Hospital D) to 42 % (Hospital B), see Table 1. Only 14 % of mother-infant pairs were prescribed regimens that were consistent with WHO 2010 Option A guidelines which requires AZT commencing at 14 weeks gestation. An urban hospital (Hospital C) evidenced the highest rate of adherence to WHO 2010 Option A guidelines (28 %) while the other three sites experienced lower rates of adherence.

### Trends in PMTCT Administration

**Fig. 2:**
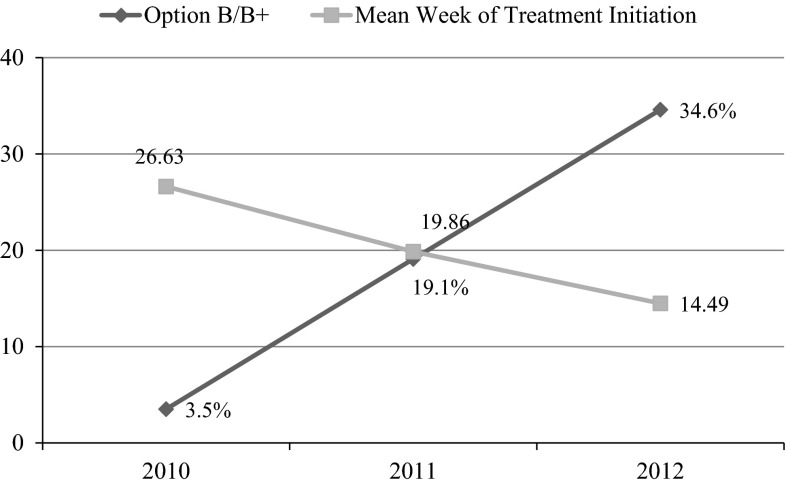
Trends in PMTCT Guideline implementation in Kenya: Mean week of PMTCT treatment initiation by year and uptake (%) of WHO Option B/B+ (2010–2012). Changes between each year: 2010–2011, 2011–2012, and 2010–2012 are statistically significant (p < 0.001) for annual increases in Option B/B+ initiation and annual decreases in mean gestational week at treatment initiation

Mean week of PMTCT treatment initiation among mothers decreased from a high of 26.6 (SD 6.2) in 2010 to 19.9 (SD 11.34) in 2011 to 14.5 (SD 11.72) in 2012, as presented in Fig. 2. A one-way ANOVA demonstrated a significant decrease in the mean week of treatment initiation over the 3 years [F(2, 1131) = 91.346, p < 0.001], and post hoc comparisons (Tukey HSD test) confirmed significant differences between each year, all p < 0.001, (Table 2). Similarly, the proportion of pairs receiving WHO Option B/B+ was calculated across hospitals for each year; resulting in <5 % (CI 1.4–5.6 %) in 2010, 19.1 % (CI 16.2–22.0 %) in 2011 and 34.6 % (CI 29.6–39.6 %) by the end of 2012. Chi squared tests confirmed significant differences in WHO Option B/B+ uptake between 2010 and 2011, χ^2^(2) = 21.10, p < 0.001, 2011 and 2012, χ^2^(2) = 36.69, p < 0.001, and 2010 and 2012, χ^2^(2) = 79.25, p < 0.001 (Fig. 2).

**Table 2:** Post-hoc analyses for changes in mean week of treatment initiation by year

Gestational year	N	Mean	Standard deviation	Standard error	95 % CI	
					Lower bound	Upper bound
2010	257	26.63	6.22	0.388	25.87	27.39
2011	585	19.86	11.34	0.469	18.94	20.78
2012	292	14.49	11.72	0.686	13.14	15.84
Total	1134	20.01	11.32	0.336	19.35	20.67

	SS	df	F	p
Between groups	20,186.841	2	91.346	0.000
Within groups	124,972.052	1131		
Total	145,158.893	1133		

### Pediatric HIV Transmission

**Fig. 3:**
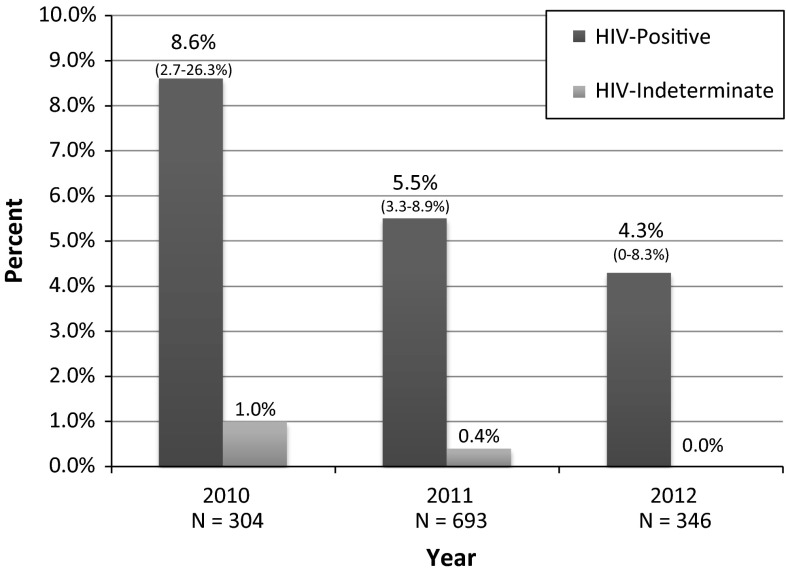
HIV+ DNA PCR test results among HIV-exposed infants enrolled in EID by year, % (range between hospitals), 2010–2012

The average pediatric HIV transmission rate over the study period was 5.9 % (CI 4.6–7.2 %), ranging from a low of 2.5 (CI 1.2–3.8 %, Hospital D) to a high of 9.9 % (CI 6.4–13.4 %, Hospital C); both located in Nairobi (Table 1). Pediatric HIV transmission rates across hospitals decreased each year; 2010 8.6 % (CI 5.4–11.7 %), 2011 5.5 % (CI 3.8–7.2 %) and 2012 4.3 % (CI 2.4–6.8 %) (Fig. 3). MTCT was significantly lower among women receiving PMTCT in 2012 compared to women receiving PMTCT in 2010 (8.6 vs. 4.3 %; χ^2^(2) = 5.02, p = 0.025). Rates of indeterminate HIV test results were consistently low across all sites with an average of 0.4 %, range 0–1.3 %.

### Predictors of Pediatric HIV Transmission

Table 3 documents variation in HIV transmission rates according to PMTCT regimen prescribed to mother-infant pairs. Women who were prescribed any PMTCT regimen (n = 1123) were significantly less likely to transmit HIV compared to those not prescribed PMTCT (n = 214; 3.6 % [CI 2.6–4.8 %] vs. 18.2 % [CI 13.6–23.9 %], χ^2^(1) = 51.62, p < 0.001). However, HIV transmission rates did not vary significantly by PMTCT regimen (i.e., late initiation 3.1 %; CI 2.1–4.6 %, WHO Option A 4.5 %; CI 2.2–9.0 %, or Option B/B+ 4.6 %; CI 2.5–8.2 %, χ^2^(2) = 1.52, p = 0.467).

**Table 3:** Likelihood of HIV transmission compared by PMTCT intervention: Infants with positive HIV DNA PCR test results among the 1337 HIV-exposed infants with conclusive PCR test results at four hospitals

	A (n = 202)	B (n = 307)	C (n = 281)	D (n = 547)	Total (n = 1337)	χ^2^	p value
Mother received PMTCT							
No PMTCT (n = 214)	18 (24.3 %)	13 (13.5 %)	7 (30.4 %)	1 (4.8 %)	39 (18.2 %)	χ^2^(1) = 51.62	p < 0.001
Mother on any PMTCT regimen (n = 1123)	1 (0.8 %)	5 (2.4 %)	21 (8.1 %)	13 (2.5 %)	40 (3.6 %)		
ART regimen							
Late AZT initiation: AZT starting at 15 weeks gestation or later (n = 749)	1/69 (1.4 %)	1/78 (1.3 %)	9/142 (6.3 %)	12/460 (2.6 %)	23/749 (3.1 %)	χ^2^(2) = 1.52	p = 0.467
Early AZT initiation: AZT starting 0–14 weeks gestation (WHO Option A) (n = 155)	0/27 (0 %)	0/46 (0 %)	7/70 (10.0 %)	0/12 (0 %)	7/155 (4.5 %)		
WHO Option B/B+ (n = 219)	0/32 (0 %)	4/87 (4.6 %)	5/46 (10.9 %)	1/54 (1.6 %)	10/219 (4.6 %)		

Denominators in the ART regimen section of this table represent the total number of mothers to receive the aforementioned PMTCT regimen at that particular hospital

The sum of all the denominators plus the number of mothers that did not receive PMTCT (n = 214) equals 1337

Total of 1337 includes all confirmed PCR results, thus excludes indeterminate (n = 6) or missing (n = 4) results

Numerators are the number of positive infants by regimen and hospital. The sum of the numerators in the ART regimen section will equal the number of mothers on any PMTCT regimen

Any PMTCT (vs. none), mixed feeding method, and hospital where care was received were significantly associated with pediatric transmission in bivariate analyses (p < 0.05). In multivariable analyses (Table 4), HIV-exposed infants who did not receive PMTCT and those who received mixed feeding were significantly more likely to be HIV+ (AOR 4.6 [2.49, 8.62], p < 0.001, and AOR 5.0 [2.77, 9.02], p < 0.001), respectively. HIV-exposed infants seeking care from a high volume yet low resource health facility (Hospital C) were three times more likely to test HIV+ compared to HIV-exposed infants receiving care at the other facilities (AOR 3.0 [1.51, 5.92], p = 0.002).

**Table 4:** Bivariate and multivariable logistic regression analyses to identify predictors of pediatric HIV transmission among mother-infant pairs enrolled in EID

	Bivariate	Multivariable
	OR [95 % CI], p	AOR [95 % CI], p
No PMTCT	6.0 [3.78, 9.65], p < 0.001	**4.6 [2.49, 8.62], p** **<** **0.001**
PMTCT regimen		
Late initiation	0.662 [0.310, 1.41], p = .286	
WHO Option A	0.989 [0.368, 2.66], p = .982	
WHO Option B (reference)		
Hospital		
Hospital A	3.9 [1.92, 7.95], p < 0.001	1.55 [0.672, 3.57], p = .304
Hospital B	2.3 [1.15, 4.78], p = 0.019	0.67 [0.279, 1.61], p = .369
Hospital C	4.1 [2.11, 7.86], p < 0.001	**3.0 [1.51, 5.92], p** **=** **0.002**
Hospital D (reference)		
Infant feeding		
Replacement feeding	0.254 [0.035, 1.86], p = 0.178	0.303 [0.040, 2.26], p = 0.244
Mixed feeding	7.43 [4.36, 12.63], p < 0.001	**5.0 [2.78, 9.02], p** **<** **0.001**
Breastfeeding (reference)		

Bold values are statistically significant (p < 0.05)

## Discussion

These findings illustrate a promising trend towards the earlier administration of more efficacious ARV PMTCT regimens; with statistically significant improvements achieved each year between 2010 and 2012. Coverage of PMTCT was high at 83 % (range 63–96 %), yet still shy of the 90 % target set by WHO [23]. Late PMTCT implementation (average of 28 weeks) was high when averaged across sites (66.3 %), while uptake of the 2010 WHO Option A and Option B/B+ regimens (by 14 weeks) was low; 14.1 and 19.6 % respectively. While Kenya is definitely moving in the right direction, challenges to the full implementation of the 2010 and more aggressive 2012 WHO PMTCT guidelines persist.

Despite less than optimal adoption of the latest WHO PMTCT guidelines, pediatric HIV transmission decreased significantly each year between 2010 and 2012 at these four government hospitals. The transmission across study hospitals in 2012 was 4.3 % (0–8.3 %), which meets the national and global target of <5 % MTCT. In fact, this rate is comparable to that (4.1 %) achieved by a formal programmatic intervention that provided adherence and retention support throughout the PMTCT cascade conducted in Malawi [25]. This 4.3 % average observed across study hospitals is significantly lower than the 2013 national Kenyan average of 14.3 % MTCT [24], which includes a more representative denominator of all HIV-exposed infants; rather than only those engaged in care. This clinic-based sample of mother-infant pairs engaged in EID likely under represents the highest-risk mothers and infants most vulnerable to transmission.

In contrast to our expectations and the existing literature evaluating PMTCT treatment regimens, [26–28] the earlier administration of more rigorous PMTCT regimens was not significantly associated with decreased infant HIV transmission when analyzed at the individual level. It’s possible that these data on initial regimen and timing of initiation, which were recorded by healthcare providers in clinical records, were not a perfect reflection of what mothers really ended up taking during their pregnancies. While the significant association between hospital and infant transmission rates cannot be explained by data collected in this study, we hypothesize that findings may be attributable to site-specific conditions like the varying PMTCT staff training, quality of patient records and PMTCT registry data, frequency of test kit and medication stock outs, and HIV prevalence among communities utilizing respective hospitals. The referral maternity hospital that draws patients from a much wider catchment area observed the lowest transmission (2.5 %), while a high volume health center located in an urban slum observed the highest transmission (9.9 %); both in the same city. Our findings emphasize the importance of facility-related variables to maximizing the benefit of PMTCT interventions.

The gap between initial administration of a PMTCT regimen and the daily events that occur before, during, and after delivery are significant. These data highlight the importance of measuring outcomes across the complete PMTCT cascade [29] including appointment attendance, medication adherence (pharmacy refill records, self-report adherence and viral load), viral resistance to current ART regimens, and retention throughout pregnancy and the postnatal period. Data from neighboring Tanzania finds that <10 % of mothers maintained at least 80 % adherence to medications during the pre, intra- and postpartum periods [19]. In Kenya, nearly one-third of pregnant women on ART in facilities in western Kenya leave care prior to delivery [30]. Consequently, progress made by initiating rigorous regimens early in the gestational period may become squandered without adequate support systems for adherence and retention; posing more complicated and long term challenges.

These data demonstrate the pace of policy implementation after the 2010 WHO PMTCT guidelines at four hospitals in Kenya and are linked to EID outcomes; the ultimate evaluation of PMTCT effectiveness. While these data provide timely and valuable insights regarding progress made toward national and global targets for eliminating MTCT, they should be interpreted in light of several limitations. Data in this sample are limited to HIV-infected mothers who presented with their infants for EID, which is estimated at 66 % of all HIV-exposed infants in a recent meta-analysis among 11 sub-Saharan countries [31]. Consequently, this study may reflect PMTCT data from healthier and more empowered women with live births. Data were gathered from medical chart review for those who received ANC at the same hospital they were seeking EID, however; for the few mothers without an existing antenatal record (<5 %), self-reported maternal history was used. Data were limited by the completeness and accuracy of clinical records and PMTCT registries which may under or over- estimate the proportion of women who received some degree of PMTCT. Importantly, these data reflect provider administration of a PMTCT regimen rather than patients’ actual medication adherence or retention in services. Consequently, it cannot be determined if PMTCT medications were taken consistently, correctly, or at all. Furthermore, at the time of this study, demographic variables such as education level and socioeconomic status were not routinely recorded in the HITSystem; limiting analyses for predictors of PMTCT uptake or perinatal HIV transmission. These data only include gestational period through the end of 2012 and thus do not capture improvements made in 2013 or beyond.

## Conclusions

Kenya continues to improve PMTCT services by administering more efficacious ARV regimens earlier in the antenatal period. These improvements are verified by annual declines in pediatric HIV transmission between 2010 and 2012. Significant differences in infant HIV transmission among hospitals and the lack of association between rigor of PMTCT regimen and pediatric HIV transmission data, illuminate that proper administration of PMTCT regimens is only one of several critical steps in reducing vertical transmission. Measuring medication adherence and retention throughout the PMTCT cascade of care are essential to understanding the real impact of PMTCT efforts.
